# Examining how topicality impacts pronoun resolution in second language processing

**DOI:** 10.3389/fnhum.2024.1456178

**Published:** 2024-11-13

**Authors:** Tingting Wang, Utako Minai, Alison Gabriele

**Affiliations:** ^1^Department of Communication Sciences and Disorders, University of Iowa, Iowa City, IA, United States; ^2^Department of Linguistics, University of Kansas, Lawrence, KS, United States

**Keywords:** second language processing, bilingualism, referential processing, Mandarin Chinese, pronoun resolution, subjecthood, topic, givenness

## Abstract

In research on second language (L2) processing, the processing of reference has been highlighted as a domain of particular difficulty, but the source of the difficulty is not well understood. The present study examines whether differences in the pronominal systems of the first language (L1) and L2 impact processing. We take a novel approach, testing a group of intermediate-advanced L2 learners in both their L1 (Mandarin Chinese) and L2 (English), allowing us to directly examine whether L2 learners show similar or different patterns when processing the L1 and L2. We also test a group of L1 English speakers. The study focused on two topicality-related factors, subjecthood and pronominalization, that have been shown to increase the prominence of an entity in the discourse, making it more likely that an entity in subject position (subjecthood) or an entity that has been referred to with a pronoun (pronominalization) will be considered as an antecedent for a subsequent pronoun. We developed a picture verification task with visual-world eye-tracking in both English and Chinese. This task provides a measure of both pronoun interpretation and online processing. Results showed subtle differences in how subjecthood and pronominalization are weighted in English and Chinese as L1s: pronominalization played a stronger role in L1 Chinese than in L1 English both in the interpretation measure and in the eye-movement data. The results for the L2 English learners showed an interesting pattern in which their results were more similar to the L1 English results on the measure of pronoun interpretation, but were more similar to the L1 Chinese results in the eye-movement data. These results show successful use of discourse cues in L2 pronoun interpretation but differences between L1 and L2 speakers during processing. It is proposed that decreased sensitivity to morphosyntactic information that is not present in the L1 (case on pronouns) leads to differences in L2 referential processing, in line with proposals that L2 learners face challenges with integrating different kinds of linguistic information online, particularly morphosyntactic information.

## 1 Introduction

The present study examines what factors impact referential processing in a second language (L2). It has been proposed that during language processing, readers and listeners construct mental models of the characters and events in a discourse (Bransford et al., 1972; van Dijk and Kintsch, 1983; Johnson-Laird, 1983), allowing them to track the relevant entities. When a new referent such as pronoun (e.g., *he*, *she*, *it*) is encountered, it can then be linked with a discourse entity that has been previously mentioned and is represented in the mental model. Not all entities are equally likely to be considered as antecedents for a reduced form such as a pronoun. As compared to a more explicit form such as a noun phrase (*the dog*) or a proper name, pronouns are more likely to refer to discourse entities that are salient or more prominently represented in the discourse model (Ariel, 1990; Givón, 1983; Gundel et al., 1993; Sanford and Garrod, 1981). Research has shown that many different factors increase the prominence of a discourse entity including properties related to syntactic position, semantics, and discourse coherence (e.g., Arnold, 1998; Arnold et al., 2000; Carminati, 2002; Garnham, 2001; Kehler, 2002; Kehler et al., 2008). The present study builds on work by Kaiser (2011) which focuses on two specific factors, subjecthood and givenness, which have been shown to increase discourse salience and are both related to the concept of being a “topic,” which is what a sentence is about (Lambrecht, 1994; Reinhart, 1981).

Discourse entities which are topics are often realized in subject position (Reinhart, 1981) and, in languages like English, it is well-established that entities appearing in subject position such as *Jamie* in (1) are more likely to be considered as the antecedent for an ambiguous (subject) pronoun such as *he* as compared to the entity in object position (*Roy*) (e.g., Arnold et al., 2000; Brennan, 1995; Chafe, 1976; Crawley et al., 1990; Stevenson et al., 1994).

**Table:** 


(1) Jamie hit Roy and he walked away.


The prominence of entities in subject position may be related to both syntactic prominence as well as semantic/thematic factors such as agentivity (e.g., Järvikivi et al., 2017). Languages such as English, in which topics often appear in subject position, often cannot clearly distinguish the role of subjecthood from topicality, but research by Rohde and Kehler (2014) has shown that discourse entities in the subject position in passive constructions (*Linda was amazed by Jane*), which are more likely to be topics, are even more likely to be referred to on subsequent mention with a pronoun than discourse entities in the subject position of active constructions, suggesting that topichood clearly plays a role independent of grammatical position.^1^

An additional factor that has been related to topicality and shown to increase discourse prominence is “givenness,” which is whether an entity has been previously mentioned (also called discourse-old) or has just been introduced (discourse-new) (Ariel, 1990; Prince, 1981). Entities which have been previously mentioned in the discourse are more likely to be referred to with a pronoun and thus *pronominalization* of a discourse entity can be a cue that the entity at stake is “given” in the discourse. The example in (2), from Kameyama (1996), shows the effects of multiple factors at play: the pronoun “He” in the third sentence in (2a) is more likely to refer to *Babar* both because *Babar* has been referred to in the subject position of the second sentence, and also because *Babar* has been pronominalized (*He*) in that sentence.

**Table:** 

	(2) Babar went to the bakery…
a.	*He* greeted the baker. He pointed at the blueberry pie.
b.	The baker greeted *him*. He pointed at the blueberry pie.

However, in (2b), subjecthood (*the baker*) and pronominalization (*him*-*Babar*) point to different entities in the second sentence. Kameyama showed that in (2b), the overall preferred antecedent for the subject pronoun *He* in the third sentence was *the baker*, but there was competition with *Babar*, who had been pronominalized, and thus established as “given,” which boosts the entity’s prominence (see also Kaiser, 2011).

Thus, previous research with L1 speakers has shown that multiple factors increase discourse prominence and impact pronoun resolution including both subjecthood and givenness (e.g., Chafe, 1976; Crawley et al., 1990; Kaiser, 2011; Kameyama, 1996). In the domain of L2 processing, the processing of reference has been highlighted as a domain of particular difficulty, but the specific source of the difficulty is not well understood (Cheng and Almor, 2017; Cunnings, 2017; Grüter et al., 2017; Sorace and Filiaci, 2006; Sorace, 2011; Roberts et al., 2008). One possible source of difficulty is crosslinguistic differences in the pronominal systems of the L1 and L2. In null subject languages such as Italian, where overt subjects do not need to be expressed in all contexts, a null pronoun is generally used to refer to a discourse prominent antecedent, such as an entity that is in subject position (see Carminati, 2002, 2005). In contrast, an overt pronoun would be used to refer to a less prominent discourse entity. Thus, the referents of overt pronouns in null subject languages like Italian differ from non-null subject languages. Sorace and Filiaci (2006) showed that English-speaking learners of Italian had particular difficulty with overt pronouns, using them to refer to discourse prominent antecedents, potentially due to influence from the L1. However, one challenge in drawing strong generalizations about L1 influence in the domain of reference is that the range of languages that has been investigated is somewhat limited and research has shown that there are important differences even among languages that share the null subject property (e.g., Filiaci et al., 2014). Gundel et al. (1993) showed that while null subjects were frequently used to refer to discourse prominent subject antecedents in languages like Japanese (70%) and Spanish (68%), other null subject languages like Russian (19%) and Chinese (28%) used null subjects far less to refer to discourse prominent antecedents, favoring unstressed overt subject pronouns instead. Thus, it is necessary to directly examine the linguistic properties of both the L1 and L2 to be able to draw conclusions with respect to L1 influence (see Cunnings et al., 2017; Contemori et al., 2019), an approach we take in the current study.

In addition, some L2 processing studies have suggested more general challenges in using discourse information to establish reference, regardless of the properties of the learners’ L1 (e.g., Ellert, 2013; Roberts et al., 2008). Results such as these led Sorace (2011) to propose that properties that lie at the interface of syntax and discourse may present persistent challenges in L2 acquisition, perhaps because bilinguals are less efficient at integrating multiple sources of information online. Thus, general processing difficulties may underlie difficulties in establishing referential dependencies rather than only crosslinguistic differences.

The present study addresses these issues by using eye-tracking to examine whether Mandarin Chinese-speaking learners of English can use cues such as subjecthood and pronominalization that have been shown to impact pronoun resolution in L1 speakers (e.g., Kaiser, 2011). To our knowledge, no previous L2 processing study has investigated how multiple cues related to topicality interact in the course of online processing. This approach allows us to systematically examine whether L2 learners can indeed integrate multiple sources of discourse information during processing or whether they are limited as compared to L1 speakers. We also address the issue of L1 influence, which, as we will discuss below, remains an open question in this domain. We examine the role of subjecthood and givenness in both Mandarin Chinese (as an L1) and English (tested in both L1 speakers and L2 leaners). This approach allows us to directly compare the role of these cues in the two languages in order to better understand their similarities and differences. Second, we take a novel approach by testing the L2 learners in both their L1 (Mandarin Chinese) and L2 (English) so that we can more directly evaluate to what extent the same factors impact processing in the L1 and the L2, and to investigate to what extent L2 processing is impacted by L1 influence. By examining the L1 and L2 within the same individual we are also able to avoid drawing conclusions about L2 processing by relying solely on comparisons between monolingual native speakers and bilinguals who, as Sorace points out, may differ in processing abilities (see also Hopp, 2022). In what follows, we will briefly review the most relevant literature including previous research on the role of subjecthood in pronoun resolution in L2 learners and previous research on subjecthood and givenness in English and Mandarin Chinese.

## 2 Background

### 2.1 The role of subjecthood in L2 pronoun resolution

Several previous studies have examined whether L2 learners can use subjecthood as a cue to discourse prominence in overt pronoun resolution and the results are mixed both with respect to whether the learners perform similarly to L1 speakers and whether there is evidence of L1 influence (e.g., Cunnings et al., 2017; Contemori and Dussias, 2020; Contemori et al., 2019; Ellert, 2013; Roberts et al., 2008; Santoro, 2020). Roberts et al. (2008) examined the interpretation and processing of subject pronouns in Dutch by German-speaking and Turkish-speaking learners. Dutch and German are both non-null subject languages while Turkish is a null subject language. In Turkish, an overt pronoun would generally be used to refer to a less prominent discourse antecedent, unlike Dutch and German. The experiment compared sentences in which pronoun reference could be established unambiguously through use of a number cue (e.g., 3a, English translation) with sentences in which the pronoun is potentially ambiguous (3b, English translation) as there are two possible gender-matching antecedents.

**Table:** 

	(3)
a.	The workers*_*i*_* are in the office. While Peter*_*j*_* is working, he*_*j*_* is eating a sandwich.
b.	Peter*_*i*_* and Hans*_*j*_* are in the office. While Peter is working, he*_*i/j*_* is eating a sandwich.

The results of an eye-tracking during reading experiment showed that Dutch native speakers showed the shortest fixations on the pronoun in the ambiguous condition in (3b) as compared to the unambiguous conditions. The ease of pronoun resolution in (3b) is arguably related to the fact that the discourse entity *Peter* is prominent in the discourse as he has been mentioned twice and is in the subject position of the preceding clause. However, both German-speaking and Turkish-speaking learners of Dutch showed the longest reading times in (3b) suggesting that, regardless of similarities or differences between the L1/L2, L2 learners cannot use the cues to discourse prominence in the same way as L1 speakers (see also Ellert, 2013). Roberts et al. (2008) also included an offline comprehension task that asked questions about the target sentences such as the one in (4) which directly examined pronoun interpretation.

**Table:** 


(4) A sandwich was eaten by ______.


For sentences such as (3b), L1 Dutch speakers overwhelmingly selected *Peter* as the antecedent for the pronoun, assigning reference to the discourse entity that was most prominent based on subjecthood. German-speaking learners showed similar patterns as the L1 Dutch speakers, while Turkish-speaking learners chose the local subject only half of the time. Roberts et al. (2008) attributed the observed differences in the offline task to L1 influence: in Turkish, overt pronouns generally refer to less prominent antecedents so reference to antecedents outside of the local subject position (e.g., *Hans* in 3b) is also possible. Thus, although L1 influence was not observed in the eye-tracking experiment, where both groups of learners performed differently from the L1 Dutch group, it was observed in the interpretation task.

Contemori et al. (2019) used an offline antecedent choice task to investigate L1 Spanish L2 English learners’ interpretation of overt pronouns in English and Spanish including sentences such as those in (5).

**Table:** 

	(5)
a.	Yolanda met Josefina while she was in high school.
b.	Carlos and Martin are at the office. While Carlos is working, he is eating lunch.

The results showed that Spanish-speaking learners of English successfully used the subjecthood cue and interpreted the ambiguous pronoun in (5a) as referring to *Yolanda*, similar to L1 English speakers. However, L1 and L2 speakers differed in the interpretation of (5b), which is similar to the context that Roberts et al. (2008) tested. L1 English speakers interpreted the pronoun as referring to the local antecedent (*Carlos*) 87% of the time. In contrast, L1 Spanish speakers at an intermediate level of proficiency in English selected the local antecedent only 59% of the time, allowing the overt pronoun to refer to the less prominent antecedent (*Martin*) more frequently than L1 English speakers. Similar results were also observed when an independent group of L1 Spanish speakers was tested in Spanish, suggesting that the results may be due to L1 influence. Contemori et al. (2019) also proposed that contexts such as (3b/5b) may be particularly difficult for L2 learners because the two antecedents are initially introduced into the discourse with similar salience in the coordinate phrase (*Carlos and Martin*), potentially leading to “interference” when the pronoun is encountered, making it harder for L2 learners to use the subjecthood cue successfully to assign reference to the local subject (*Carlos*).

Cunnings et al. (2017) also examined whether L2 learners of English whose L1 was a null subject language (Greek) could use subjecthood as a cue to discourse prominence in the processing of overt pronouns in a visual-world eye-tracking experiment. In the two critical conditions, shown in (6a/b), the pronoun is potentially ambiguous because there are two possible gender-matching antecedents. They created a subject versus object bias by manipulating the visual display. Specifically, once the biasing noun (*the ice cream*) was heard in the auditory stimuli, the visual display biased the interpretation by placing the ice cream next to the subject antecedent (*Peter* in 6a) or the object antecedent (*Peter* in 6b).

**Table:** 

	(6)
a.	Subject Bias, Ambiguous
	After Peter spoke to Mr. Smith by the till in the shop, he paid for the expensive ice cream that looked tasty.
b.	Object Bias, Ambiguous
	After Mr. Smith spoke to Peter by the till in the shop, he paid for the expensive ice cream that looked tasty.

Results revealed that Greek-speaking learners of English at an intermediate-advanced level of proficiency showed similar patterns as L1 English speakers by generally showing more looks to the subject antecedent until encountering the biasing noun, at which point, both groups were similarly biased by the visual display in the expected direction. This similarity between the L1 and L2 groups is different from what Roberts et al. (2008) observed in their eye-tracking study, where L2 learners were unable to use discourse cues to pronoun resolution during processing. Cunnings et al. (2017) attributed the success of the L1 Greek learners in L2 English in their eye-tracking study as compared to the L2 learners in Roberts et al. (2008) to linguistic differences between English and Dutch as Dutch has different pronominal forms that index topic maintenance (personal pronouns) and topic shift (d-pronouns) and thus, may have a more complex system of reference.

Santoro (2020) examined the interpretation of overt subject pronouns in L2 English, in this case by advanced Mandarin Chinese-speaking learners. Santoro focused on syntactic constructions in which it had been reported in the theoretical literature that using an overt pronoun in Mandarin Chinese encodes a shift in topic and thus the pronoun would be more likely to refer to a non-subject antecedent (e.g., *The more Peter explained to his father what really happened, the more relieved he felt*) (Huang, 1991). Santoro examined whether the L1 Mandarin Chinese-speaking learners of English would show L1 influence, interpreting overt pronouns in English as referring to non-subject antecedents. However, the results showed a similar subject bias in both L1 and L2 English speakers, although the L2 group was slower to respond. Santoro suggested that the advanced proficiency level of the L2 learners may have precluded the study’s ability to observe effects of L1 influence.

Overall, the results of previous studies are inconsistent with respect to whether or not L2 learners can use the subjecthood cue successfully in overt pronoun resolution and whether there is evidence of L1 influence. With respect to interpretation, Roberts et al. (2008) and Contemori et al. (2019) both observed potential effects of L1 influence in a context where two potential antecedents were introduced with similar salience (3b/5b), suggesting that learners whose L1 is a null subject language may interpret the overt pronoun as referring to a less prominent antecedent, unlike native speakers, at least in certain contexts. Santoro did not observe L1 influence in his study, an effect that he attributed to the advanced level of proficiency of the learners tested. With respect to online processing, Roberts et al. (2008) observed difficulty for L2 learners while Cunnings et al. (2017) showed similar patterns for both natives and L2 learners. It is important to point out that the cue that Cunnings et al. (2017) manipulated in their eye-tracking experiment was visual, so a further test of whether L2 learners can use linguistic discourse cues during processing is warranted.

### 2.2 Subjecthood and pronominalization in pronoun resolution in English

The present study builds directly on the first experiment in Kaiser (2011). Kaiser (2011) used a visual-world eye-tracking experiment to investigate how two topicality-related factors (subjecthood and givenness) affect pronoun interpretation in English. Givenness was tested using *pronominalization*. As discussed above, discourse entities which have been previously mentioned are more likely to be referred to with a pronoun, and thus, pronominalization has also been found to boost discourse prominence (Kameyama, 1996). The goal of Kaiser’s first experiment was to see if subjecthood and pronominalization have separable effects on pronoun interpretation and processing: the Baseline condition (7) introduced two discourse-new names in the critical sentence, one in subject position and one in object position as in (7b), followed by a “look-away clause” in (7c) which directed attention away from the characters and toward a third object in the display, and then an ambiguous pronoun in the test sentence (7d). In the Baseline condition, the most likely antecedent for the ambiguous pronoun is *Greg*, based on subjecthood.

**Table:** 

	(7) Baseline
a.	n/a (no Lead-in sentence)
b.	Greg congratulated John enthusiastically yesterday (Critical sentence).
c.	The prizes for the best-ranked tennis players were about to be announced, and (Look-away clause)
d.	he was holding a new yellow tennis racket (Test sentence).
e.	Everyone was in a good mood that day (Wrap-up).

The goal of the Pronominalized Subject condition in (8) was to see if the subject preference would be further boosted if the antecedent in subject position was additionally referred to with a pronoun, and thus indexed as discourse-old. In (8a), one character (*Greg*) is introduced into the discourse in subject position and then is pronominalized in the subject position in the critical sentence in (8b), which also introduces a character in object position. The rest of the story including the test sentence was identical to the Baseline (see 7c–d). In the Pronominalized Subject condition, both subjecthood and pronominalization cues point to the pronominalized subject (*Greg/he*).

**Table:** 

	(8) Pronominalized subject condition
a.	Greg is always very supportive of others (Lead-in sentence).
b.	He congratulated John enthusiastically yesterday (Critical sentence).

In the Pronominalized Object condition in (9), subjecthood and pronominalization are pitted against each other as they point to different entities. In (9a) one character (*Greg*) is introduced in the subject position and then is pronominalized in object position in the critical sentence (9b). The critical sentence in (9b) also introduces a character in subject position (*John*). The rest of the story was identical to the Baseline (see 7c–e). In the Pronominalized Object condition, the most recently mentioned entity in subject position is *John*, but *Greg* may also be prominent as he was pronominalized (in object position) and thus, indexed as discourse-old. Kaiser examined whether pronominalizing the object would decrease the subject antecedent preference or even lead to an object preference if pronominalization was weighted more strongly than subjecthood.

**Table:** 

	(9) Pronominalized object condition
a.	Greg did very well in last month’s tennis tournament (Lead-in sentence).
b.	John congratulated him enthusiastically yesterday (Critical sentence).

Kaiser used a picture-verification task with simultaneous eye-tracking in which L1 English speakers were instructed to identify mismatches between the story and the corresponding visual display by clicking on the region of the picture that contained the mismatch. All target items contained a mismatch. For example, the test sentence associated with the stories above mention that *he is holding a yellow tennis racket*, but in the visual display neither *Greg* nor *John* was holding a yellow tennis racket (they had rackets of different colors). By clicking on the character whom they associate with the mismatch, participants implicitly indicated their interpretation of the pronoun.

Kaiser analyzed participants’ click responses in the picture verification task as well as their eye-movements during the test sentence starting from the pronoun onset. In the picture verification task, participants showed a subject preference in all three conditions, but the Pronominalized Subject condition had more subject choices (83%) than the Baseline condition (72%), indicating a boosted subject preference when subjecthood and pronominalization cues coincide, and the Pronominalized Object condition had more object choices than the Baseline condition, indicating a weakened subject preference (62%) in the presence of a pronominalized object. Thus, with respect to interpretation, both pronominalization and subjecthood cues play a role but subjecthood had a stronger effect.

In terms of eye movements, both the Pronominalized Subject condition and Baseline condition showed a significant subject preference, with more looks to the subject in the Pronominalized Subject condition as compared to the Baseline in the final time window. However, in the Pronominalized Object condition, there was no clear preference for either the subject or the object antecedent in all time windows, indicating close competition between the subject and the pronominalized object. The eye-movement results suggest that, for L1 English, during online processing, pronominalization and subjecthood cues are equally important.

### 2.3 Pronoun resolution in Mandarin Chinese

Mandarin Chinese (henceforth Chinese) is a language that allows noun phrases to be omitted in both subject and object positions when they can be clearly understood from the context, as is shown in the short discourse in (10) which has been modified from Li and Thompson (1981, 662). Chinese is also characterized as a topic-prominent language (Li and Thompson, 1981) in which topics appear in sentence-initial position.

**Table:** 

(10)^2^					
a.	wài	biān	jìn lái le	yi	ge	rén,
	outside enter come aspect one CL person					
	“From outside came a person…					
b.	liǎng	ge	hóng	yǎnjing,		
	two	CL	red	eye		
	(He) had two red eyes…					
c.	yi	fù	dà yuán liǎn,			
	one	CL	big round face,			
	(and) one big round face,					
d.	dài	zhe	yi	ge	xiǎo	màozi,
	wear	DUR	one	CL	small	hat
	and (he) was wearing a small hat.”					
e.	tā	xìng	Xià.			
	3sg surname Xia.					
	“He had the surname Xia.”					

In (10), clauses (a–d) refer to the same referent and represent what has been called a *topic chain*, in which null pronouns are used to refer to the topic of the preceding clause (Li and Thompson, 1981). However, in (e), although the referent is maintained, an overt pronoun (*tā*) is used, showing that in Chinese, overt pronouns can be used even in contexts where the referent is prominent in the discourse. In (e), the overt pronoun is preferred because a different aspect of information about the referent is being highlighted (his name as compared to his appearance).

Experimental work on Chinese also supports the idea that null and overt pronouns have similar referential biases, and both can refer to discourse prominent antecedents such as those in subject position (Yang et al., 1999, 2001). Simpson et al. (2016) provided similar evidence related to the role of subjecthood for the interpretation of overt pronouns in Chinese. The results of a series of sentence-completion tasks showed that L1 Chinese speakers showed a preference for subject antecedents when interpreting ambiguous overt pronouns, and thus, the evidence suggests that overt pronouns clearly do not generally refer to less prominent antecedents as in other null subject languages. However, a recent study by Zhang and Kwon (2022) showed that while Chinese readers showed a preference for subject antecedents when interpreting both null and overt pronouns, such bias was stronger in null pronouns. Cui and Hwang (2023) further examined how topicality impacted the interpretation of null and overt pronouns in Chinese. In the topical condition, subjects were fronted using a left-dislocation structure (e.g., *Because Xiaoming bet Xiaoli, so*….). They found that topicality increased subject reference for both null and overt pronouns, and similar to the results in Zhang and Kwon (2022), null pronouns showed a stronger bias for subject reference (86.5%) as compared to overt pronouns (71.7%). Overall, these results suggest that while there is a similar subject bias for both null and overt pronouns in Chinese, and both are impacted by topicality, there are quantitative differences between the two referential forms.

## 3 Current study

The present study examines two main questions: (1) Do topicality-related factors such as subjecthood and givenness (pronominalization) impact the interpretation and on-line processing of pronouns in L2 English? (2) Is the interpretation and processing of pronouns in L2 English impacted by L1 influence? In order to address the first question, we test both L1 English speakers and L1 Chinese L2 learners of English to examine the role of subjecthood and pronominalization in the interpretation and processing of pronouns in both populations.

To address the second question, we take a novel approach, testing a group of intermediate-advanced L2 learners in both their L1 (Mandarin Chinese) and L2 (English). This approach allows us to (1) better understand the role of subjecthood and pronominalization in Chinese and (2) to directly compare how those cues are used when processing the L1 and L2. To our knowledge, no previous study has directly examined the role of both subjecthood and pronominalization in the interpretation and processing of overt pronouns in Chinese. While the role of subjecthood is well-established by the previous studies discussed above, whether or not subjecthood and givenness (pronominalization) are separable effects in Chinese, similar to what Kaiser (2011) found in English, remains an open question that we investigate in the present study.

In order to compare the role of subjecthood and pronominalization in L1 and L2 processing, our statistical analyses combine our three datasets (L1 English speakers, L2 English, L1 Chinese) allowing us to see directly whether the results for the L2 English speakers are more similar to the results for the L1 English speakers or whether the L2 English results more closely pattern with the results for the learners’ L1 Chinese. Building directly on the first experiment in Kaiser (2011), we developed a picture verification task with visual-world eye-tracking in both English and Chinese. This task is ideal for examining reference as it provides both a measure of online processing and an implicit measure of pronoun interpretation.

### 3.1 Participants

We recruited 49 L1 English speakers (31 female, 18 male, mean age 20.72, range 18–27) living in the United States and 50 Chinese-speaking learners of English (29 female, 21 male, mean age 27.6, range 18–50).^3^ L1 English speakers were recruited from undergraduate classes at a public university in the US. L1 Chinese participants were recruited by posting recruitment flyers through the university mailing list as well as on Chinese social networking platforms. All learners considered themselves as L1 speakers of Mandarin Chinese^4^ and reported that they started to learn English in a school setting from age 4 onward (*M* = 9.07, range: 4–22), with an average of 14.82 years of classroom study (range: 7–30); no learners reported using English at home during childhood. All Chinese participants have self-reported English proficiency scores at an intermediate or higher level, as indicated by English language assessment tests for college admission purposes (e.g., TOEFL > 90, IELTS > 7). Learners also took the University of Michigan Listening Comprehension Test, a 45-question test targeting various aspects of English grammar (e.g., question formation, verb inflections); the mean score was 83.8/100 (range: 57.4–93.6) placing learners at an intermediate-advanced level of proficiency.

### 3.2 Design and materials

#### 3.2.1 Picture verification task: English

For the picture verification task, both auditory and visual stimuli were developed. A total of 18 stories were developed with 12 of the stories being modified versions of those used in Kaiser (2011, Experiment 1).^5^ Each story had three conditions (see Table 1).

**TABLE 1:** Example stimuli: English picture verification task.

**1. Baseline condition**	
a. Lead-in sentence	n/a
b. Critical sentence	Greg congratulated John enthusiastically yesterday.
c. Look-away clause	The stage was being set up for the final group photo of the tennis tournament
d. Test sentence	and he was holding a new yellow tennis racket.
e. Wrap-up sentence	Everyone was pleased with how the season went.
**2. Pronominalized subject condition**	
a. Lead-in sentence	Greg was in a good mood.
b. Critical sentence	He congratulated John enthusiastically yesterday.
c. Look-away clause	The stage was being set up for the final group photo of the tennis tournament
d. Test sentence	and he was holding a new yellow tennis racket.
e. Wrap-up sentence	Everyone was pleased with how the season went.
**3. Pronominalized object condition**	
a. Lead-in sentence	John was in a good mood.
b. Critical sentence	Greg congratulated him enthusiastically yesterday.
c. Look-away clause	The stage was being set up for the final group photo of the tennis tournament
d. Test sentence	and he was holding a new yellow tennis racket.
e. Wrap-up sentence	Everyone was pleased with how the season went.

Across all conditions, a *Critical Sentence* included two characters of the same gender (e.g., *Greg* and *John*) in subject and object position. This sentence was followed by a *Look-away Clause* which mentioned an inanimate object in the visual display (e.g., the stage) to divert looks away from the two characters before the test sentence. The *Test Sentence* contained an ambiguous subject pronoun, and the story then concluded with a *Wrap-up Sentence*. The Pronominalized Subject (2) and the Pronominalized Object conditions (3) additionally included a *Lead-in Sentence* (2a, 3a). This sentence is required to be able to pronominalize one of the two characters in the following Critical Sentence, either in the subject position (2b) or in the object position (3b).

While we adopted Kaiser’s design, we made some modifications to the stories. First, we controlled the lead-in sentences in the Pronominalized Subject and Pronominalized Object conditions to be identical (with the exception of the names) so that the two conditions differed only in terms of which character was pronominalized. For the twelve stories that we modified from Kaiser (2011), we also revised the wording to ensure that the vocabulary was appropriate for L2 learners and that the stories followed similar plot lines.

The 18 sets of target stories were divided into three Latin-square lists such that no participant was presented with more than one story condition from the same set in the same language. In each list, we also included 30 filler stories to counterbalance stimulus presentation factors and mask the purpose of the experiment (target to filler ratio was 3:5). In the filler stories, the referent of the pronoun could be resolved using a gender cue on the pronoun. A full list of the English stimuli can be found at this link: https://osf.io/zrxbj/. The target and filler items in each list were divided into four blocks to allow three timed breaks (2 min) for each participant. After each break, participants were instructed to complete calibration again continue the experiment. The order of presentation of the items were randomized in each block.

**FIGURE 1:**
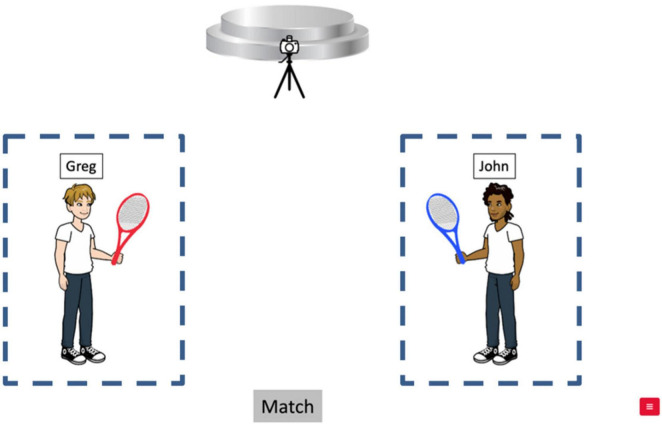
Example of the visual display for an English target trial; Areas of Interest (AOI) for the eye-movement analysis are indicated with dotted lines (not presented during testing). Figures created by the authors using the comic creation tool Pixton.

Six characters (3 male, 3 female) with gender-stereotypical names were used for all the target and filler stories. 24 out of 30 fillers included different-gender characters, while 6 had same-gender characters, ensuring equal representation of gender combinations across all stimuli. All stories in the English task were recorded by a female native speaker with an American midwestern accent. She was instructed not to place any phonological prominence on the pronoun while reading. The audio was normalized for amplitude and processed using *Praat* (Boersma, 2001; Boersma and Weenink, 2024). Each story was associated with a unique visual display consisting of two characters identified by written names and a “look-away object” associated with the inanimate objects (e.g., *the stage*) mentioned in the look-away sentence in the story (see Figure 1). All characters were created using the online comic creation tool *Pixton*^6^ ; the inanimate objects were created using clipart available online. A “Match” button was located at the bottom of the display in the middle of the screen. Across items, each character appears three times in both audio and visual stimuli, counterbalanced with respect to the order of mention in the auditory story (NP1 vs. NP2) and the spatial locations on the screen (Left vs. Right side).

Participants were instructed to listen to the story while viewing the visual display and judge whether the picture matched or mismatched with the story. If there was a match between the story and the visual display, participants were instructed to click on the “Match” icon; if there was a mismatch, participants were instructed to click on the part of the visual display that presented a mismatch. Crucially in all 18 target items, the test sentence presented a mismatch with respect to the color of an item associated with the characters. For example, in all three conditions in Table 1, the test sentence includes the phrase *and he was holding a new yellow tennis racket* but in the corresponding visual display (Figure 1), neither character is holding a yellow tennis racket. Thus, “clicks” on a character in the visual display, which indicate the source of the mismatch (e.g., he is holding a red racket, not a yellow one), provide an implicit measure of how the pronoun is interpreted. The task allows us to see the participant’s antecedent choice for each item without the need to explicitly ask about the pronoun. Among the 30 fillers, 24 stories presented a “match” and 6 had a mismatch with respect to the inanimate object mentioned in the look-away clause, balancing the total ratio of matching/mismatching items to 1:1.

#### 3.2.2 Picture verification task: Chinese

We developed 18 sets of Chinese stories based on the English stories described above as well as 30 filler stories. The content of the stories was modified to ensure maximum naturalness (e.g., placement of adverbs differs in the two languages) and cultural appropriateness in Chinese. Table 2 shows a target story example in each of the three conditions. A full list of the Chinese stimuli can be found at this link: https://osf.io/zrxbj/.

**TABLE 2:** Example stimuli: Chinese picture verification task.

**1. Baseline condition**	
a. Lead-in	n/a
b. Critical sentence	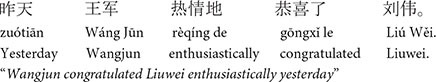
c. Look-away Clause	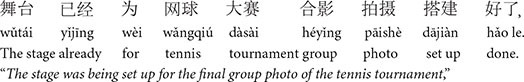
d. Test sentence	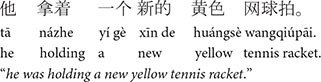
e. Wrap-up sentence	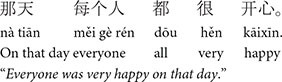
**2. Pronominalized subject condition**	
a. Lead-in sentence	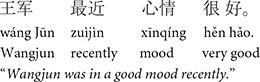
b. Critical sentence	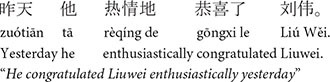
c. Look-away clause	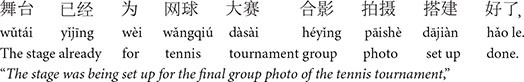
d. Test sentence	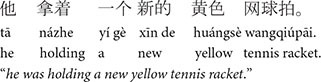
e. Wrap-up	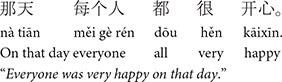
**3. Pronominalized object condition**	
a. Lead-in	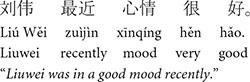
b. Critical sentence	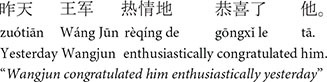
c. Look-away clause	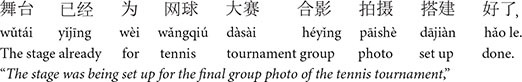
d. Test sentence	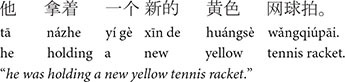
e. Wrap-up sentence	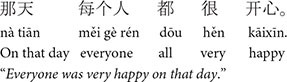

In Mandarin Chinese, third person pronouns do not encode gender or case information in the spoken form. In the auditory stimuli, all forms of the pronouns (subject and object) were pronounced as *ta* (although the written form distinguishes between the male “他” and female “她” genders).

The 18 sets of stories were divided into three Latin-square lists such that no participant was presented with more than one story condition from the same set. We ensured that participants did not see the same condition from each story in English and Chinese by giving participants different lists in each language. Six characters (3 male, 3 female) with gender-stereotypical names were used for all the target and filler stories. Chinese audio stimuli were recorded by a female native speaker of Mandarin Chinese. The audio was normalized for amplitude and processed using *Praat* (Boersma, 2001; Boersma and Weenink, 2024).

The visual displays for the Chinese task were identical to those used in the English version, with the exception of the Chinese names. The six characters were introduced as a group of international students studying in China, each with a Chinese name.

### 3.3 Procedures

The experiment was conducted remotely during the pandemic via the online testing platform *Gorilla Experiment Builder* (Anwyl-Irvine et al., 2020). The picture verification task was conducted with simultaneous eye-tracking using Gorilla’s *Eye Tracking Zone* which utilizes *Webgazer.js* (Papoutsaki et al., 2016). This eye-tracking system detects participants’ faces through their webcams and estimates their eye-gaze locations on the screen in real time relying on prediction models. Prior to the task, participants’ eye-movements were calibrated using a 5-point calibration option. At the beginning of the session, participants provided their consent to participate in the study and met with the experimenter on Zoom for a preliminary check of their settings. This included verifying the functionality of their webcams and audio, as well as optimizing their remote eye-tracking setup, which included checking their lighting, location and position of sitting, and distance from the computer. Participants then were given a short introduction to the task. Following this brief session, participants exited Zoom and completed the session independently.

During the picture verification task, each trial began with a fixation cross (700 ms) in the center of the screen, followed by a silent picture display (1000 ms), and then the audio presentation started along with the picture display remained on the screen. Participants were instructed to wait until the end of the story to make their judgment (Match/Mismatch). Calibration was repeated before each block of the task to ensure accurate eye gaze capture by the webcam. Participants were asked to take four two-minute breaks during completion of the picture verification task. Eye-movement data were collected during the auditory presentation of the story, but we only analyzed the data collected during the duration of the test sentence (Table 1, *d* sentences).

The English native speakers completed the English picture verification task in one session. Chinese-speaking participants completed the English and Chinese versions of the picture verification task across two different sessions separated by at least one week, first completing the task in English, and then in Chinese. Following the English task, L2 learners also completed the English proficiency task. As our main research interest was to examine the possible influence of the learners’ L1 (Chinese) on their L2 (English) we decided to test all learners in their L2 (English) first (as opposed to counterbalancing the order) to ensure that previous testing in the L1 would not influence the L2 results. All participants received monetary compensation for their participation.

### 3.4 Predictions

Our first research question asks whether topicality-related factors such as subjecthood and givenness (pronominalization) impact the interpretation and online processing of pronouns in L2 English. If Chinese L2 English speakers are able to evaluate discourse prominence using both subjecthood and pronominalization, we expect to observe the same patterns in both L1 and L2 English speakers. Specifically, in line with Kaiser (2011), in the Baseline condition [see Table 1, (1)], the discourse entity in subject position (e.g., *Greg*) is most prominent and thus is the most likely antecedent for the pronoun. In the Pronominalized Subject condition [Table 1, (2)], both subjecthood and pronominalization point to the same discourse entity (e.g., *Greg*) and thus, there may be an additional boost in prominence for the subject as compared to the Baseline. In the Pronominalized Object condition [Table 1, (3)], subjecthood (e.g., *Greg*) and pronominalization (e.g., *John*) point to different discourse entities, and thus there may be competition between them, particularly in the eye-tracking results.

However, previous L2 studies have generally only reported similar patterns for L1 speakers and L2 learners when the contexts introduced two antecedents, in the subject and object position, respectively, and discourse prominence was largely determined only by subjecthood (e.g., Contemori et al., 2019; Contemori and Dussias, 2020; Cunnings et al., 2017; Santoro, 2020). In other studies, where the two potential antecedents were introduced with similar salience (see 3b/5b), L2 learners have shown difficulty (e.g., Contemori et al., 2019; Roberts et al., 2008). In addition, L2 studies which have looked at other discourse cues such as event structure have shown a lack of sensitivity (Grüter et al., 2017). If L2 learners struggle to evaluate discourse prominence when multiple cues are relevant, or if they struggle to use the pronominalization cue, which has not been examined in previous L2 studies, they may show a greater reliance on the subjecthood cue, showing a similar subject preference in all three conditions.

Our second research question asks if the interpretation and processing of pronouns in L2 English is impacted by L1 influence. To answer this question, we first need to understand how L1 Chinese speakers evaluate discourse prominence using subjecthood and pronominalization. As discussed above, there is ample evidence that subjecthood plays a role in Chinese (e.g., Simpson et al., 2016; Yang et al., 1999, 2001; Zhang and Kwon, 2022) and thus, we predict that L1 Chinese speakers will also show a subject preference in the Baseline condition (Table 2). There is good reason to expect that pronominalization will also impact pronoun resolution because, as was discussed above, pronouns in Chinese often refer to topics, and thus, pronominalization is likely to boost discourse prominence. However, the weighting of these two cues is unclear as this question has not been examined in previous studies. If subjecthood plays a stronger role than pronominalization, we may observe an overall subject preference across conditions, with pronominalization modulating the extent of the preference, as was observed in Kaiser (2011) results for the English picture verification task. If subjecthood and pronominalization play roughly equal roles, we may observe a boosted subject preference in the Pronominalized Subject condition (where the cues point to the same discourse entity), and competition between the subject and object in the Pronominalized Object condition (where the cues point to different discourse entities), as was observed in Kaiser’s English eye-movement data. If pronominalization plays a stronger role than subjecthood, we will observe an object preference in the in the Pronominalized Object condition (where the cues point to different discourse entities).

With respect to L1 influence, after we are able to establish what patterns hold in L1 Chinese, our analysis will allow us to examine whether the learners in L2 English more closely resemble L1 speakers of English or whether the L2 English data looks more similar to the L1 Chinese data. We will also be able to determine if L1 influence plays a role in both the picture verification task, which examines pronoun interpretation (as in Roberts et al., 2008; Contemori et al., 2019), and during online processing.

### 3.5 Data processing: picture verification task

#### 3.5.1 Picture verification data

For each trial, we recorded participants’ clicks on either one of the two characters (left/right), on the inanimate third object, or on the “Match” button.

#### 3.5.2 Eye-movement data

Because we used a web-based eye-tracking system which is less reliable due to the use of inconsistent devices across participants and the need to rely on the internet to record eye movements, we were not able to follow Kaiser (2011) in analyzing the proportion of fixations to the subject vs. object referents dynamically on a millisecond by millisecond timeline. Instead, we examine the overall proportion of fixations to the subject vs. object referents during the presentation of the target sentence.

The recorded eye-movement data included participants’ eye gaze locations estimated on the coordinates of pre-set zones containing the two characters, which we defined as the Areas of Interest (AOIs) (Figure 1) during the auditory presentation of the Test sentence (e.g., Table 1, 1d). For each AOI, raw and normalized pixel coordinates were provided in the data files, with normalized coordinates based on the window sizes of participants’ devices. We used the normalized coordinates in calculating the boundaries (left, right, top and bottom) of the two AOIs.^7^ The data files also included predicted gaze locations in pixels and normalized pixels, recorded at varying time intervals due to variability in prediction generation.^8^ Each predicted gaze location accompanied a “face_conf” value, indicating how strongly the image under the model resembles a face. The values range from 0 to 1, 0 indicating no fit and 1 indicating perfect fit. Following *Gorilla*’s recommendations, values over 0.5 indicate a good fit.

The normalized gaze coordinates (x and y) were compared to the boundaries of two AOIs during the *Test Sentence* time-window. If the eye gaze fell within the boundaries of either one of the AOIs, it was recorded and associated with that AOI. The process was conducted using the “add_aoi” function in the *eyetrackingR* package (Dink and Ferguson, 2015) in R (R Core Team, 2021). We then conducted two data pre-processing steps to ensure that we included only validated eye data in the analysis. First, empty target trials with no recorded eye data during the test sentence presentation were identified. The empty trials were likely due to unstable internet connection during the experiment. Five English native speakers had over 50% of empty target trials and thus their eye-movement data was excluded from the analysis. Second, we excluded eye data with a “face_conf” value below 0.5, as discussed above. After the two steps, 2.85% of the eye-data for L1 English speakers, 5.15% of the eye-data for learners in the English task, 5.28% of the eye-data for learners in the Chinese task were excluded. Third, in order to isolate only those eye-movements associated with processing during the presentation of the test sentence (Table 1, *d* across conditions) we removed eye-movement data that was recorded either before the onset of the pronoun or after the offset of the test sentence in the audio presentation. To align the eye-movement data recording with the pronoun onset, we measured the duration of the word *and* in the audio recording, and excluded data recorded during its presentation. After these data processing steps, 666,964 datapoints from the three groups were entered into the model for statistical analysis.

## 4 Results

### 4.1 Picture verification data

**FIGURE 2:**
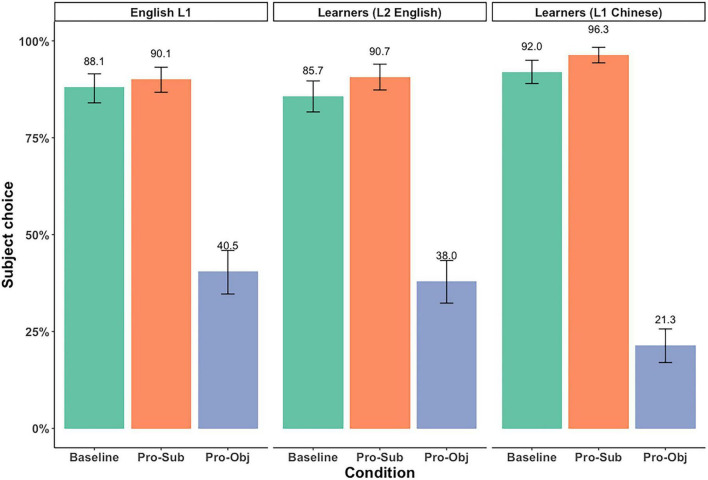
Mean proportion of subject choice in the picture verification task.

The mean proportions of subject antecedent choice across the three conditions in both the English and Chinese picture verification task are summarized in Figure 2, and Table 3.^9^ We present results for the L1 English speakers and the L2 learners who took the task in both L2 English and their L1 Chinese.^10^

**TABLE 3:** Mean percentage of subject and object choices.

Group	Condition	Subject (%)	Object (%)
L1 English	Baseline	88.10	11.90
	Pro-Sub	90.14	8.50
	Pro-Obj	40.48	58.84
Learners: L2 English	Baseline	85.67	8.00
	Pro-Sub	90.67	4.33
	Pro-Obj	38.00	56.67
Learners: L1 Chinese	Baseline	92.00	6.33
	Pro-Sub	96.33	2.00
	Pro-Obj	21.33	77.67

Following Kaiser (2011), we first used one-group *t*-tests to examine whether the proportion of subject choices was significantly above chance level (50%) in all three conditions.^11^ Results of the one-group *t*-tests showed that, for L1 English speakers, and for L2 learners in both L1 Chinese and L2 English, the proportion of subject choices was significantly above chance level in the Baseline and Pronominalized Subject Condition, and significantly below chance level in the Pronominalized Object Condition, in both the by participants and by items analysis (statistical results available at: https://osf.io/zrxbj/).

Next, we used Mixed-effects logistic regression models in comparing the subject choice in the Pronominalized Subject condition and the Pronominalized Object condition, in comparison to the Baseline to examine whether the same degree of subject preference held across the three conditions for the following three sets of data (“*Group*”): data collected from L1 English speakers (L1 English); data collected from Chinese-speaking learners in the English task (Learners: L2 English); data collected from Chinese-speaking learners in the Chinese task (Learners: L1 Chinese). The dependent variable was *Subject choice* (1 = subject choice, 0 = non-subject choice). Fixed effects were *Condition* (Baseline, Pronominalized Subject, Pronominalized Object, dummy coded^12^) and *Group* (L1 English, Learners: L2 English, Learners: L1 Chinese, sum-coded).^13^ Each model included *Participant* and *Item* as random effects. All models contained the maximal random effects structure (Barr et al., 2013) by including a by-participant slope for *Condition*, and a by-item slope for *Group*. A by-participant slope for *Condition*, and a by-item slope for Condition*Group were initially included but then excluded to allow the model to converge. The parameter estimates of the model are listed in Table 4.

**TABLE 4:** Results of the mixed-effects logistic models for the picture-verification data.

Predictors	Estimate	Std. error	z value	Pr(> |z|)
**Number of obs: 2,682, participant: 99, item: 54**				
(Intercept)	2.25	0.17	13.55	< 0.001***
Condition (Pro-Sub)	0.55	0.23	2.35	0.02*
Condition (Pro-Obj)	−3.07	0.21	−14.85	< 0.001***
Group1 (L1 English)	−0.09	0.17	−0.52	0.60
Group2 (Learners in L2)	−0.30	0.15	−2.03	0.04*
ConditionPro-Sub:Group1	−0.31	0.23	−1.32	0.19
ConditionPro-Obj:Group1	0.46	0.19	2.49	0.01*
ConditionPro-Sub:Group2	−0.01	0.23	−0.06	0.95
ConditionPro-Obj:Group2	0.56	0.18	3.12	0.00**

Model formula: *glmer*(Subject choice ∼ Condition*Group+(1| Participant)+(1| Item)).

*p <0.05;

**p < 0.01;

***p < 0.001.

The results showed a significant effect of *Condition (Pro-Sub)*, showing that the subject choices *increased* significantly in the Pronominalized Subject Condition compared to the Baseline Condition, and the trend was observed among all three groups. There was also a significant effect of *Condition (Pro-Obj)*, suggesting that the subject choices were *decreased* in the Pronominalized Object condition compared to the Baseline condition. The effect of *Condition (Pro-Obj)* also significantly interacted with *Group1*, suggesting that the decreased subject choices in the Pronominalized Object Condition is smaller for the L1 English speakers, compared to the average of all three groups, as indicated by the positive estimate (ß = 0.46). There was also a significant *Condition (Pro-Obj)* by *Group2* interaction indicating that the decreased subject effect is also smaller for the L2 English learners, compared to the average of all three groups (ß = 0.56).

In order to further examine the interactions with *Condition (Pro-Object)*, we conducted a follow-up analysis. The same Mixed-effects logistic regression analysis was conducted, but we changed the fixed effect *Group* into dummy coding and set the reference group to *Learners in L2 English*. This allowed us to directly examine whether the patterns of the L2 English learners were more similar to the L1 English patterns or the L1 Chinese patterns. A by-participant slope for *Condition*, and a by-item slope for Condition*Group were initially included but were excluded to solve the convergence issue and the singular fit warning. The optimizer “bobyqa” was also included to allow the model to converge. The parameter estimates of the model are listed in Table 5.

**TABLE 5:** Results of the mixed-effects logistic models for the picture-verification data (follow-up analysis).

Predictors	Estimate	Std. error	z value	Pr(> |z|)
**Number of obs: 2,682, participant: 99, item: 54**				
(Intercept)	1.95	0.21	9.07	< 0.001***
Condition (Pro-Sub)	0.53	0.30	1.77	0.08.
Condition (Pro-Obj)	−2.50	0.26	−9.60	< 0.001***
Group (Learners in L1)	0.70	0.27	2.56	0.01*
Group (L1 English)	0.22	0.27	0.80	0.42
Condition (Pro-Sub):Group (L1 English)	−0.30	0.37	−0.79	0.43
Condition (Pro-Obj):Group (L1 English)	−0.10	0.31	−0.33	0.74
Condition (Pro-Sub):Group (Learners in L1)	0.33	0.46	0.74	0.46
Condition (Pro-Obj):Group (Learners in L1)	−1.58	0.33	−4.75	0.00***

Model formula: *glmer*(Subject choice ∼ Condition*Group+(1| Participant)+(1| Item)).

*p <0.05;

**p < 0.01;

***p < 0.001.

First, there was a marginally significant *Condition (Pro-Sub)* effect for the reference group (Learners in L2 English), suggesting that the increased subject choices by the L2 English learners in the Pronominalized Subject condition from the Baseline condition was weaker when the L2 English data were considered independently. Second, there was a significant *Condition (Pro-Obj)* effect and a significant interaction between *Condition (Pro-Obj)* and *Group (Learners in L1 Chinese)*. The simple effect of *Condition (Pro-Obj)* means that the reference group (Learners in L2) showed significantly decreased subject choices in the Pronominalized Object condition. The interaction indicates that the L1 Chinese results showed a bigger decrease in subject choices as compared to the L2 English results. The lack of a significant interaction between *Condition (Pro-Obj)* and *Group (L1 English)* suggests that the degree of the decreased subject choices in the Pronominalized Object condition is similar between the L1 English speakers and the L2 English learners. Thus, the main takeaway from the pronoun interpretation data is that L2 English learners were more similar to the L1 English speakers and that the L2 learners showed different patterns in the Pronominalized Object condition in their L1 Chinese and in the L2 English.

### 4.2 Eye-movement data: results

**FIGURE 3:**
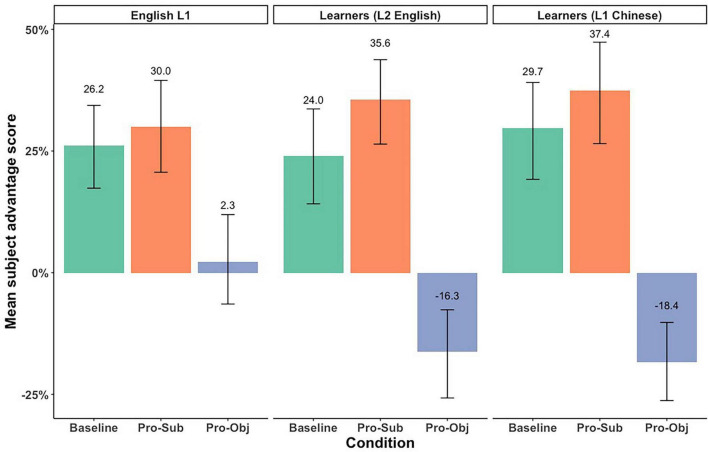
Subject advantage scores in the eye-movement data.

We followed Kaiser (2011) in first comparing the proportion of subject looks relative to the object looks using paired *t*-tests to determine if the subject preference reached significance within each participant group. We also calculated the *Subject Advantage Scores* for each trial by each participant, by subtracting the proportion of looks to the object from the proportion of looks to the subject. The mean subject advantage scores are summarized in Figure 3.

Results of paired *t*-tests showed that all three Groups looked at the subject significantly more than the object in the Baseline and Pronominalized Subject conditions. However, in the Pronominalized Object condition, L1 English speakers did not show any significant differences between looks to the subject and object (Subject: 50.99%, *p* = 0.63), while learners looked at the object significantly more than the subject in both the L2 English (Object: 56.81%) and in L1 Chinese (Subject: 60.11%) (Table 6). Details of the statistical results are available at: https://osf.io/zrxbj/.

**TABLE 6:** Mean proportion of looks to the subject and object during the test sentence.

Group	Condition	Subject (%)	Object (%)
L1 English	Baseline	62.98	37.02
	Pro-Sub	65.32	34.68
	Pro-Obj	50.99	49.01
Learners: L2 English	Baseline	63.86	36.14
	Pro-Sub	67.62	32.38
	Pro-Obj	43.19	56.81
Learners: L1 Chinese	Baseline	65.11	34.89
	Pro-Sub	70.28	29.72
	Pro-Obj	39.89	60.11

In order to compare the degree of subject bias in participants’ eye-movements in the three conditions during the presentation of the test sentence, mixed-effects linear regression models were conducted, including *Subject Advantage Scores* as the dependent variable, and *Condition* (Baseline, Pronominalized Subject, Pronominalized Object, dummy coded), *Group* (L1 English, Learners: L2 English, Learners: L1 Chinese, sum coded) and the interaction between them as fixed effects. Additionally, all models included *Participant* and *Item* as random effects and satisfied the maximal random effects structure (Barr et al., 2013) by including a by-participant slope for *Condition*, and a by-item slope for *Group*, but then excluded to allow the model to converge. The parameter estimates of the model are listed in Table 7.

**TABLE 7:** Results of the mixed-effects linear regression models for the eye-movement data.

Predictors	Estimate	Std. Error	*t* value	Pr(> |t|)
**Number of obs: 2,370, participant: 94, item: 54**				
(Intercept)	0.28	0.03	10.84	0.00***
Condition (Pro-Sub)	0.08	0.03	2.20	0.03*
Condition (Pro-Obj)	−0.39	0.03	−11.21	0.00***
Group1 (L1 English)	−0.02	0.03	−0.56	0.58
Group2 (Learners in L2)	0.00	0.03	−0.09	0.93
Condition (Pro-Sub):Group1	−0.03	0.05	−0.61	0.54
Condition (Pro-Obj):Group1	0.15	0.05	3.08	0.00**
Condition (Pro-Sub):Group2	0.00	0.05	0.02	0.99
Condition (Pro-Obj):Group2	−0.03	0.05	−0.59	0.56

Model formula: *lmer*(Subject advantage score ∼ Condition*Group+(1| Participant)+1| Item).

*p <0.05;

**p < 0.01;

***p < 0.001.

Results showed a significant effect of *Condition (Pro-Sub)*, suggesting a significant increased subject advantage in participants’ eye looks in the Pronominalized Subject condition compared to the Baseline, when combining all three groups. Results also revealed a significant effect of *Condition (Pro-Obj)* and a significant interaction between *Condition (Pro-Obj)* and *Group1 (L1 English)*. These results suggest that while all three Groups on average showed a decreased subject advantage in the Pronominalized Object condition compared to the Baseline, L1 English speakers had a smaller decrease than the average of all three Groups, as indicated by the positive estimate in the interaction effect (ß = 0.15).

In order to further examine the interaction effect that emerged with *Condition (Pro-Obj)* we conducted a follow-up analysis, changing the fixed effect *Group* to dummy coding and setting the reference group as *Learners in L2*. This allowed us to directly examine whether the patterns of the L2 English learners were more similar to the L1 English patterns or the L1 Chinese patterns. A by-participant slope for *Condition*, and a by-item slope for Condition*Group were initially included but then excluded to allow the model to converge and avoid the singular fit warning. The parameter estimates of the model are listed in Table 8.

**TABLE 8:** Results of the mixed-effects linear regression models for the eye-movement data (follow-up analysis).

Predictors	Estimate	Std. error	*t* value	Pr(> |t|)
**Number of obs: 2,370, participant: 94, item: 54**				
(Intercept)	0.28	0.04	6.53	0.00***
Condition (Pro-Sub)	0.08	0.06	1.33	0.18
Condition (Pro-Obj)	−0.41	0.06	−7.21	0.00***
Group (Learners in L1)	−0.02	0.06	−0.28	0.78
Group (L1 English)	0.03	0.06	0.45	0.65
Condition (Pro-Sub):Group (L1 English)	−0.03	0.08	−0.37	0.71
Condition (Pro-Obj):Group (L1 English)	0.17	0.08	2.12	0.03*
Condition (Pro-Sub):Group (Learners in L1)	0.03	0.08	0.34	0.73
Condition (Pro-Obj):Group (Learners in L1)	−0.09	0.08	−1.14	0.25

Model formula: *lmer*(Subject advantage score ∼ Condition*Group+(1| Participant)+(1| Item)).

*p <0.05;

**p < 0.01;

***p < 0.001.

In this model, the *Condition (Pro-Sub)* effect was not significant, since the effect was examined only within the reference group (Learners in L2). There was a significant *Condition (Pro-Obj)* effect indicating that L2 English learners showed a significant decreased subject advantage in the Pronominalized Object condition as compared to the Baseline, and a significant interaction between *Condition (Pro-Obj)* and *Group (L1 English)* interaction indicating that L1 English speakers exhibited a weaker decrease in the subject advantage in the Pronominalized Object condition compared to the L2 English learners. The absence of a significant interaction between *Condition (Pro-Obj)* and *Group (Learners in L1 Chinese)* suggests that learners showed a similar degree of decreased subject advantage in the Pronominalized Object condition in L1 Chinese and L2 English. The most important takeaway from the eye-movement data is that patterns observed in the L2 English learners were more similar to the patterns observed in L1 Chinese than the patterns observed for L1 English, particularly for the Pronominalized Object condition.

### 4.3 Summary of results

A summary of the most important findings is presented in Table 9.

**TABLE 9:** Mean percentage of the subject choices in the picture verification data and mean proportion of looks to the subject in the eye-movement data.

Group	Condition	PVT: Subject choice (%)	Proportion of looks to Subject: (%)
L1 English	Baseline	88.10	62.98
	Pro-Sub	90.14	65.32
	Pro-Obj	40.48	50.99
Learners: L2 English	Baseline	85.67	63.86
	Pro-Sub	90.67	67.62
	Pro-Obj	38.00	43.19
Learners: L1 Chinese	Baseline	92.00	65.11
	Pro-Sub	96.33	70.28
	Pro-Obj	21.33	39.89

For the picture verification data, all three groups showed a similar increase in the percentage of subject choices in the Pronominalized Subject condition as compared to the Baseline. This effect was weaker when the data were examined only within the reference group (L2 English learners). A group difference emerged in the Pronominalized Object condition: L1 Chinese speakers showed a greater decrease in subject choices from the Baseline as compared to the L2 English learners, who exhibited a pattern similar to that of L1 English speakers. This was indicated by the absence of a significant interaction between Condition (Pro-Obj) and Group (L1 English) in the follow-up analysis. Overall, while all groups showed a boosted subject bias in the Pronominalized Subject condition and an object bias in the Pronominalized Object condition, the object bias was stronger in L1 Chinese.

For the eye-movement data, all three groups showed a similar increase in the number of looks to the subject in the Pronominalized Subject condition as compared to the Baseline. This effect was not significant when the data were examined only within the reference group (L2 English learners). A group difference emerged again in the Pronominalized Object condition, but with a different pattern from the picture verification results: L1 English speakers showed a smaller decrease in subject choices from the Baseline compared to L2 English learners who showed more looks to the object, similar to the pattern observed in L1 Chinese. This was indicated by the absence of a significant interaction between Condition (Pro-Obj) and Group (Learners in L1 Chinese) in the follow-up analysis. In sum, for the Pronominalized Object condition, L1 English speakers show equal competition between the subject and object antecedents while the learners showed an object bias in both L1 Chinese and L2 English.

## 5 Discussion

The present study examined two main questions: (1) Do topicality-related factors such as subjecthood and pronominalization impact the interpretation and processing of pronouns in L2 English? (2) Is the interpretation and processing of pronouns in L2 English impacted by L1 influence? The results presented an interesting picture in which the L2 English learners clearly used the subjecthood and pronominalization cues similarly to L1 English speakers on the picture verification task, but with respect to the eye-movement data, the L2 English learners showed similar patterns in L1 Chinese and L2 English. We will first discuss the interesting differences that emerged in L1 English and L1 Chinese so that we can discuss the L2 English patterns against that background.

For the L1 English speakers, in both the picture verification data and in the eye-movement data, there was a subject bias in the baseline and subject choice was further boosted when the subject was also pronominalized. Different results emerged on the two measures in the Pronominalized Object condition, where subjecthood and pronominalization were pitted against each other. Results for the picture verification task showed an object bias, suggesting that pronominalization was weighted more heavily than subjecthood, but in the eye-movement data, the two cues were weighted equally as the proportion of looks to the subject and object were roughly equal.

Our results differed from Kaiser (2011) on the picture verification task in that we observed a stronger subject bias in the baseline (88% in our study as compared to 72% in Kaiser, 2011), and an object bias in the Pronominalized Object condition (58.84% looks to the object) as compared to a subject bias (62% looks to the subject) in Kaiser’s results. Although we followed Kaiser’s approach closely, we did revise the English stimuli, and it is possible that the revisions to the stories (including controlling the lead-in sentence across conditions) led to these different patterns. Nevertheless, it is important to highlight that the eye-movement results were very similar in the two studies, and we replicated Kaiser’s finding that subjecthood and pronominalization were in clear competition in the Pronominalized Object condition when the two cues pointed to different discourse entities. Thus, although our use of a web-based eye-tracking system did not allow us to look at the eye-movement data dynamically over time as in Kaiser (2011), we replicated the main pattern of results.

The results for L1 Chinese were interesting in that the biases we observed were more categorical than the ones observed for English, and similar patterns emerged in both the picture verification data and in the eye-movement data. There was a strong subject bias in the Baseline condition in line with previous studies (e.g., Simpson et al., 2016; Yang et al., 1999, 2001). The subject effect was further boosted in the Pronominalized Subject condition, despite the strong subject bias in the Baseline. It is possible that the subject bias is so strong because Mandarin Chinese is a topic-prominent language in which topics appear in initial position (Li and Thompson, 1981). In the Baseline condition (see Table 2), the subject is in the initial position and in the Pronominalized Subject condition, the subject character is referred to in the initial position twice. While the study does not allow us to tease apart subjecthood and topichood (e.g., Rohde and Kehler, 2014; Cui and Hwang, 2023), the discourse structure of Chinese may be responsible for the strong subject bias. In the Pronominalized Object condition, when subjecthood and pronominalization were pitted against each other, pronominalization was weighted more heavily, and there was a strong object bias. Multiple factors may have led to this bias. First, in the Pronominalized Object condition, a character is introduced first in the lead-in sentence as a topic, and is then pronominalized in object position in the Critical sentence, which signals that the character is “given” in the discourse, further boosting its salience. The morphosyntactic form of the pronoun may also be relevant. Third person singular pronouns in spoken Chinese do not encode case information so the object pronoun in the Critical sentence and the subject pronoun in the Test sentence are both pronounced as *ta*. Previous studies suggest that it is natural to use an overt pronoun in Chinese when continuing to refer to the same prominent character when a different aspect of the character is being highlighted or when there has been a subtle shift in time, location, or action (Li and Thompson, 1981; Simpson et al., 2016). In Chinese, it seems that the most natural interpretation of the story is that the two overt pronouns are linked to the same antecedent. A similar kind of “discourse continuity” effect has been discussed by Hwang (2023) and Hwang and Lam (2023) in studies that showed that Chinese and Korean speakers tend to use null pronouns to signal continuity of both the subject (referent continuity) and the actions in an event (action continuity). They argue that reduced expressions such as pronouns may signal continuity in discourse. The shared morphological form of the subject and object pronouns in Chinese may also contribute to the strong bias for the two overt pronouns to be interpreted as referring to the same object antecedent.

In contrast, in L1 English, it is possible that an object bias is not observed to the same extent in the Pronominalized Object condition because the subject pronoun in the Test sentence is marked for case (e.g., *he*: nominative case). Although the pronominalized object is prominent in the discourse, there is more competition from the subject in the Critical sentence possibly because there is a preference for pronouns to take antecedents with parallel grammatical roles (Smyth, 1994). This may have overridden the “discourse continuity” effect that we observed in Chinese and may have led to the competition we observed in the English eye-movement data where subjecthood and pronominalization were weighted equally. Thus, we believe that differences in discourse structure and in morphosyntax may explain the differences that emerged in English and Chinese as L1s.

With that linguistic background in mind, we turn to the results for the L2 English learners. The first research question asked if subjecthood and pronominalization impact the interpretation and processing of pronouns in L2 English. On the picture verification task, the L2 English results closely matched those of the L1 English speakers. The boost in subject preference in the Pronominalized Subject condition was only marginal when the L2 learners were examined in isolation but the pattern was very similar in the two groups. In the Pronominalized Object condition, the pattern observed for the L2 English learners was more similar to the pattern observed in L1 English than in L1 Chinese. While all three datasets showed an object bias, there was a much stronger object bias in L1 Chinese. These results show that the L2 learners are clearly distinguishing the L1 Chinese and the L2 English in pronoun interpretation. A strength of the approach that we took is that we can directly observe this distinction for the same individuals. Thus, the L2 learners were clearly successful in using discourse cues such as subjecthood and pronominalization to resolve ambiguous pronouns. These results show that difficulty with integrating discourse information is not inevitable, in line with previous L2 studies on pronoun interpretation (e.g., Contemori et al., 2019; Santoro, 2020), but our results additionally showed that L2 learners can weight multiple cues such as subjecthood and pronominalization similarly to L1 speakers.

Nevertheless, we observed differences between the L2 English learners and the L1 English speakers in the eye-movement data, particularly in the Pronominalized Object condition [see (3) in Table 2]. In this case, while L1 English speakers showed competition between the subject and object antecedents during processing, weighting subjecthood and pronominalization similarly, the L2 English learners weighted pronominalization more strongly than subjecthood, showing an object bias. There was also an object bias in L1 Chinese and the statistical analyses of the eye-movement data showed that the L2 learners showed similar patterns in L2 English and L1 Chinese. Thus, at first glance, the simplest interpretation is that during processing, L2 learners show evidence of L1 influence, weighting subjecthood and pronominalization similarly in both languages, and potentially not distinguishing between the L1 and L2. This interpretation would suggest, in response to our second research question, that there is L1 influence during processing, but not in pronoun interpretation, the opposite of the pattern that was observed in Roberts et al. (2008). However, one piece of evidence that calls this interpretation into question is that L2 English learners showed an object bias in the Pronominalized Object condition in *both* the picture verification task (where they more similar to L1 English speakers) and in the eye-movement data (where the patterns were more similar to L1 Chinese). Thus, importantly, their own weighting of the cues in L2 English is consistent across the two measures.

A second account that we want to consider is that the L2 learners differ from L1 English speakers during processing because they are less sensitive to the case information on the subject pronoun in the test sentence in L2 English. In the Pronominalized Object condition, we proposed for L1 English speakers that although the pronominalized object is prominent in the discourse (as is shown in the object bias in the results of the picture verification task), during processing, there is competition between the subject and object antecedents because the pronoun in the test sentence bears nominative case. However, if the L2 learners are less sensitive to case information, as has been observed in previous studies with learners whose L1 does not mark case (e.g., Hopp, 2010; Frenck-Mestre et al., 2018), then there will be less competition from the subject antecedent. If this interpretation is right, it seems that the L2 English learners ultimately arrive at the same interpretation as L1 English speakers (as was shown on the picture verification task), but take a somewhat different route during processing. Hopp (2022) points out that a key question for L2 processing research should be whether processing is guided by the constraints of the L2 learners’ grammar. We believe that this may be a case in which referential processing is guided by the constraints of the learners’ interlanguage grammar, which may show variability with respect to case marking. Although we do not have an independent measure of the learners’ sensitivity to case either offline or online, the learners in our study are at an intermediate-advanced proficiency level, and thus, a reduced sensitivity to case during processing would be in line with previous studies such as Hopp (2010) who showed that while near-native speakers processed case inflection similarly to native speakers, advanced learners did not.

This second account also assumes a role for L1 influence, but in the morphosyntactic domain, not in the weighting of the discourse cues. The difference between the two interpretations is that on the first account, L2 learners simply process the L2 English as if it is the L1 Chinese, weighting the cues identically during processing (but then ultimately distinguishing pronouns in the two languages in interpretation). According to the second account, L2 learners do weight the cues similarly in the two languages during processing, but for different reasons. In L1 Chinese, pronominalization is weighted more strongly because it is natural to interpret the two pronouns as referring to the same prominent object antecedent. In L2 English, pronominalization is weighted more strongly than subjecthood because the L2 learners are less sensitive to case marking on the subject pronoun and thus, there is less competition from the subject antecedent. While both accounts are plausible, we believe that the second is more likely. If the L2 English learners were simply processing the L2 similarly to the L1 Chinese, we believe that the object bias in the picture verification task should be stronger in L2 English. The fact that L2 learners distinguished L1 Chinese and L2 English in pronoun interpretation at least suggests that the languages were also treated distinctly during processing. Examining L1 Chinese learners who are at a higher level of proficiency (and thus may have more reliable knowledge of case marking) or examining L2 learners whose L1 marks case on pronouns would help to shed light on this issue.

Overall, we believe that our findings are in line with Sorace (2011) proposal that difficulties in L2 processing are often due to challenges in integrating different kinds of linguistic information. However, as Sorace also discusses, it is not that linguistic phenomena which entail the integration of discourse information are inevitably difficult. This study showed that L2 learners can successfully use discourse cues to resolve pronouns, even weighting those cues similarly to native speakers in interpretation. Differences between L1 and L2 speakers emerged during processing, which is evidence in support of Sorace’s proposal that L2 performance is often impacted by task conditions (see also Hopp, 2007, 2011). Our examination of L2 processing was limited in that our use of a web-based eye tracker only allowed us to examine the overall proportions of looks to the subject/object antecedents, as opposed to being able to track the dynamics of processing over time. Nevertheless, the measure was sensitive enough to capture differences between L1 and L2 speakers. We argued that those differences were mostly likely due to L2 learners’ difficulty integrating morphosyntactic information related to case (for related discussion see Hopp, 2009). This proposal is also in line with Slabakova (2008), who proposed that functional morphology is a bottleneck in L2 acquisition and that difficulties in the morphosyntactic domain can impact acquisition in other linguistic domains, such as meaning. We believe that this is an interesting avenue for future studies on L2 referential processing to pursue.

## Data Availability

The raw data supporting the conclusions of this article will be made available by the authors, without undue reservation.

## Footnotes

1 See Rizzi (2005, 2018) for formal analyses of the distinction between subjects and topics. He argues that topics can only be used when a connection to the discourse background can be established but subjects do not have this requirement.

2 In Li and Thompson (1981), the following abbreviations were used in the glossing: PFV, perfective aspect; CL, classifier; DUR, Durative aspect; 3sg, third-person singular.

3 40 learners were living the US, 1 in Canada, 2 in Australia, 2 in Singapore, 1 in France and 4 in China. The L2 learners who did not live in countries where English is a majority language were retained in the analysis because they were at a similar level of English proficiency as the other learners (see also footnote 9). More detailed background information is available on OSF: https://osf.io/zrxbj/.

4 Some participants also spoke another dialect of Chinese, including Sichuan (*n* = 4), Wu (*n* = 3), Cantonese (*n* = 2), Hunan (*n* = 2), Shanghai (*n* = 2), Dongzhi (*n* = 1), Jiangsu (*n* = 1), Jishou (*n* = 1), Shandong (*n* = 1), Taiwanese (*n* = 1), Hokkien (*n* = 1).

5 We removed four stories from Kaiser’s (2011) stimuli that were either hard to depict in pictures or used verbs that may not be as familiar to learners (e.g., the stories included the verbs *scold*, *scratch*, *tickle*, and *slap*) and developed six new stories (using the verbs *annoy*, *call*, *greet*, *hug*, *meet*, and *visit*).

6
www.pixton.com

7 Even though the coordinates were normalized across different window sizes, small differences in the coordinates started to occur if looking at the fourth or fifth significant figure (within 1%). In order to take into account those small differences across participants, we took the mean of the normalized coordinates of the two AOIs of each participant.

8 WebGazer.js did not provide a consistent sampling rate since there is a slight variable delay in generating predictions based on the participant’s computer and browser. On average, a sample was taken every 10 ms, with a 3 ms variability, or occasionally a longer interval once every 100 or so trials.

9 In some conditions, the proportions of clicks on the subject and on the object do not add up to 100% because participants also clicked on the Match button.

10 A reviewer pointed out that five of the L2 participants in our sample live in countries where English is not a majority language (four live in China and one in France), and these participants may show different patterns than the L2 learners who live in an immersion environment. We conducted additional analyses excluding these participants and the main patterns of results across all analyses remained the same (The additional analyses are available on OSF: https://osf.io/zrxbj/). Additionally, these participants are at the same level of English proficiency as the other participants in our study and thus, to increase our sample size, we included them in our analysis. Addressing a similar point, a second reviewer asked if the four participants who live in China showed different patterns in Chinese from the remaining participants who do not live in an environment of Chinese immersion. We conducted additional analyses excluding these participants and the main patterns of results remained the same. In addition, we did not observe large differences in Chinese proficiency, as measured by the Chinese LexTALE, between the participants who lived in China (Mean = 79, SD = 4) and those who didn’t (Mean = 77, SD = 4).

11 We used one group *t*-tests to see if our results replicated those of Kaiser (2011). Additional analyses using mixed effects logistic regression showed the same pattern of results.

12 We used dummy coding for the fixed effect *Condition*, as it compares subject choices in the Pronominalized Subject and Object conditions with those in the reference level, the Baseline, which aligns with our research interests. Specifically, 0 = Baseline; 1 = Pronominalized Subject; 1 = Pronominalized Object.

13 We used sum coding for the variable *Group*, since we are interested in whether any differences in subject choices between conditions hold across all three groups (e.g., an interaction between Condition and Group). Specifically, L1 English is coded as (1, 0), Learners in L2 English is coded as (0, 1) and Learners in L1 Chinese was coded as (−1, −1). In the results of the statistical models, Group 1 refers to L1 English L1, and Group 2 refers to L2 English learners.

## References

[B1] Anwyl-Irvine A. L. Massonnié J. Flitton A. Kirkham N. Evershed J. K. (2020). Gorilla in our midst: An online behavioral experiment builder. *Behav. Res. Methods* 52 388–407. 10.3758/s13428-019-01237-x 31016684 PMC7005094

[B2] Ariel M. (1990). *Accessing noun-phrase antecedents (rle linguistics b: Grammar).* Milton Park: Routledge.

[B3] Arnold J. E. (1998). *Reference form and discourse patterns*. [Ph.D. thesis]. Stanford, CA: Stanford University.

[B4] Arnold J. E. Eisenband J. G. Brown-Schmidt S. Trueswell J. C. (2000). The rapid use of gender information: Evidence of the time course of pronoun resolution from eyetracking. *Cognition* 76 B13–B26. 10.1016/S0010-0277(00)00073-1 10822045

[B5] Barr D. J. Levy R. Scheepers C. Tily H. J. (2013). Random effects structure for confirmatory hypothesis testing: Keep it maximal. *J. Mem. Lang.* 68 255–278. 10.1016/j.jml.2012.11.001 24403724 PMC3881361

[B6] Boersma P. (2001). Praat, a system for doing phonetics by computer. *Glot. Int.* 5 341–345.

[B7] Boersma P. Weenink D. (2024). *Praat: Doing phonetics by computer [Computer program]. Version 6.4.13.* Available online at: http://www.praat.org/ (accessed June 1, 2020)

[B8] Bransford J. D. Barclay J. R. Franks J. J. (1972). Sentence memory: A constructive versus interpretive approach. *Cogn. Psychol.* 3 193–209. 10.1016/0010-0285(72)90003-5

[B9] Brennan S. E. (1995). Centering attention in discourse. *Lang. Cogn. Process.* 10 137–167. 10.1080/01690969508407091

[B10] Carminati C. (2005). Processing reflexes of the feature hierarchy (Person > Number > Gender) and implications for linguistic theory. *Lingua* 113 259–285. 10.1016/j.lingua.2003.10.006

[B11] Carminati M. N. (2002). *The processing of Italian subject pronouns*. [Ph.D. thesis]. Amherst, MA: University of Massachusetts.

[B12] Chafe W. (1976). “Givenness, contrastiveness, definiteness, subjects, topics, and point of view,” in *Subject and topic*, ed. Li C. N. (Cambridge, MA: Academic Press), 25–55.

[B13] Cheng W. Almor A. (2017). The effect of implicit causality and consequentiality on nonnative pronoun resolution. *Appl. Psycholinguist.* 38 1–26. 10.1017/S0142716416000035

[B14] Contemori C. Dussias P. (2020). The processing of subject pronouns in highly proficient L2 speakers of English. *Glossa* 5:38. 10.5334/gjgl.972

[B15] Contemori C. Asiri O. Irigoyen E. D. P. (2019). Anaphora resolution in L2 English: An analysis of discourse complexity and cross-linguistic interference. *Stud. Second Lang. Acquisit.* 41 971–998. 10.1017/S0272263119000111

[B16] Crawley R. A. Stevenson R. J. Kleinman D. (1990). The use of heuristic strategies in the interpretation of pronouns. *J. Psycholinguist. Res.* 19 245–264. 10.1007/BF01077259 2231480

[B17] Cui W. Hwang H. (2023). “The influence of topicality on interpretation of null and overt pronouns in Mandarin,” in *Paper presented the architectures and mechanisms for language processing Asia 2023*, (Hong Kong).

[B18] Cunnings I. (2017). Parsing and working memory in bilingual sentence processing. *Bilingualism Lang. Cogn.* 20 659–678. 10.1017/S1366728916000675

[B19] Cunnings I. Fotiadou G. Tsimpli I. (2017). Anaphora resolution and reanalysis during L2 sentence processing: Evidence from the visual world paradigm. *Stud. Second Lang. Acquisit.* 39 621–652. 10.1017/S0272263116000292

[B20] Dink J. W. Ferguson B. (2015). *eyetrackingR: An R library for eye-tracking data analysis.* Available online at: http://www.eyetrackingr.com (accessed June 28, 2021).

[B21] Ellert M. (2013). Information structure affects the resolution of the subject pronouns er and der in spoken German discourse. *Discours* 12:56. 10.4000/discourse.8756

[B22] Filiaci F. Sorace A. Carreiras M. (2014). Anaphoric biases of null and overt subjects in Italian and Spanish: A cross-linguistic comparison. *Lang. Cogn. Neurosci.* 29 825–843. 10.1080/01690965.2013.801502

[B23] Frenck-Mestre C. Kim S. Y. Choo H. Ghio A. Herschensohn J. Koh S. (2018). Look and listen! The online processing of Korean case by native and non-native speakers. *Lang. Cogn. Neurosci.* 34 385–404. 10.1080/23273798.2018.1549332

[B24] Garnham A. (2001). Mental models and the interpretation of anaphora. *Psychol. Press*. 10.4324/9780203782873

[B25] Givón T. (1983). “Topic continuity in discourse: An introduction,” in *Topic continuity in discourse: A quantitative cross-language study*, ed. Givón T. (Amsterdam: John Benjamins Publishing), 1–42.

[B26] Grüter T. Rohde H. Schafer A. J. (2017). Coreference and discourse coherence in L2: The roles of grammatical aspect and referential form. *Linguist. Approach. Bilingual.* 7 199–229. 10.1075/lab.15011.gru 33486653

[B27] Gundel J. K. Hedberg N. Zacharski R. (1993). Cognitive status and the form of referring expressions in discourse. *Language* 85 274–307. 10.2307/416535

[B28] Hopp H. (2007). *Ultimate attainment at the interfaces in second language acquisition: Grammar and processing*. [Ph.D. thesis]. Groningen, MA: University of Groningen.

[B29] Hopp H. (2009). The syntax–discourse interface in near-native L2 acquisition: Off-line and on-line performance. *Bilingual. Lang. Cogn.* 12 463–483. 10.1017/S1366728909990253

[B30] Hopp H. (2010). Ultimate attainment in L2 inflection: Performance similarities in non-native and native speakers. *Lingua* 120 901–931. 10.1016/j.lingua.2009.06.004

[B31] Hopp H. (2011). Extended patterns and computational complexity. *Linguist. Approach. Bilingual.* 1 43–47. 10.1075/lab.1.1.04hop 33486653

[B32] Hopp H. (2022). Second language sentence processing. *Annu. Rev. Linguist.* 8 235–256. 10.1146/annurev-linguistics-030821-054113

[B33] Huang Y. (1991). A neo-Gricean pragmatic theory of anaphora. *J. Linguist.* 27 301–335. 10.1017/S0022226700012706

[B34] Hwang H. (2023). The influence of discourse continuity on referential form choice. *J. Exp. Psychol. Learn. Mem. Cogn.* 49 626–641. 10.1037/xlm0001166 36006717

[B35] Hwang H. Lam S. Y. (2023). “The influence of action continuity on reference form in Mandarin and English,” in *Poster presented at 36th annual conference on human sentence processing*, (Pittsburgh, PA: University of Pittsburgh).

[B36] Järvikivi J. van Gompel R. P. Hyönä J. (2017). The interplay of implicit causality, structural heuristics, and anaphor type in ambiguous pronoun resolution. *J. Psycholinguist. Res.* 46 525–550. 10.1007/s10936-016-9451-1 27629115

[B37] Johnson-Laird P. N. (1983). *Mental models: Towards a cognitive science of language, inference, and consciousness (No. 6).* Cambridge, MA: Harvard University Press.

[B38] Kaiser E. (2011). Focusing on pronouns: Consequences of subjecthood, pronominalisation, and contrastive focus. *Lang. Cogn. Process.* 26 1625–1666. 10.1080/01690965.2010.523082

[B39] Kameyama M. (1996). “Indefeasible semantics and defeasible pragmatics,” in *Quantifiers, Deduction and context*, eds Kanazawa M. Pinon C. de Swart H. (Stanford, CA: CSLI), 111–138. 10.48550/arXiv.cmp-lg/9506016

[B40] Kehler A. (2002). *Coherence, reference, and the theory of grammar.* Stanford, CA: CSLI Publications.

[B41] Kehler A. Kertz L. Rohde H. Elman J. L. (2008). Coherence and coreference revisited. *J. Semant.* 25 1–44. 10.1093/jos/ffm018 22923856 PMC3424618

[B42] Lambrecht K. (1994). *Information structure and sentence form: Topic, focus, and the mental representations of discourse referents*, Vol. 71. Cambridge: Cambridge university press, 10.1017/CBO9780511620607

[B43] Li C. N. Thompson S. A. (1981). *Pronouns in discourse: Mandarin Chinese: A functional Reference grammar.* Berkeley, CA: University of California Press, 657–675.

[B44] Papoutsaki A. Laskey J. Huang J. (2016). “WebGazer: Scalable webcam eye tracking using user interactions,” in *Proceedings of the 25th international joint conference on artificial intelligence (IJCAI)*, (New York, NY), 1839–3845.

[B45] Prince E. F. (1981). “Toward a taxonomy of given-new information,” in *Radical pragmatics*, ed. Cole P. (New York, NY: Academic Press), 223–233.

[B46] R Core Team (2021). *R: A language and environment for statistical computing.* Vienna: R Foundation for Statistical Computing.

[B47] Reinhart T. (1981). Pragmatics and linguistics: An analysis of sentence topics. *Philosophica* 27:5869. 10.21825/philosophica.82606

[B48] Rizzi L. (2005). “On some properties of subjects and objects,” in *Proceedings of the XXX incontro di grammatica generativa*, eds Brugé L. Giusti G. Munaro N. Schweikert W. Turano G. (Venezia: Cafoscarina), 203–224.

[B49] Rizzi L. (2018). “Subjects, topics and the interpretation of pro,” in *From sounds to structures: beyond the veil of maya*, eds Petrosino R. Cerrone P. van der Hulst H. (Berlin: De Gruyter Mouton), 510–530. 10.1515/9781501506734-019

[B50] Roberts L. Gullberg M. Indefrey P. (2008). Online pronoun resolution in L2 discourse: L1 influence and general learner effects. *Stud. Second Lang. Acquisit.* 30 333–357. 10.1017/S0272263108080480

[B51] Rohde H. Kehler A. (2014). Grammatical and information-structural influences on pronoun production. *Lang. Cogn. Neurosci.* 29 F912–F927. 10.1080/01690965.2013.854918

[B52] Sanford A. J. Garrod S. C. (1981). *Understanding written language: Explorations of comprehension beyond the sentence.* Hoboken, NJ: John Wiley & Sons Ltd.

[B53] Santoro M. (2020). The acquisition of English anaphoric expressions by adult Chinese speakers. *J. Psycholinguist. Res.* 49 641–662. 10.1007/s10936-020-09717-4 32623565

[B54] Simpson A. Wu Z. Li Y. (2016). Grammatical roles, coherence relations, and the interpretation of pronouns in Chinese. *Lingua Sin.* 2 1–20. 10.1186/s40655-016-0011-2

[B55] Slabakova R. (2008). *Meaning in the second language.* Berlin: Mouton de Gruyter.

[B56] Smyth R. (1994). Grammatical determinants of ambiguous pronoun resolution. *J. Psycholinguist. Res.* 23 197–229. 10.1007/BF02139085

[B57] Sorace A. (2011). Pinning down the concept of “interface” in bilingualism. *Linguist. Approach. Bilingual.* 1 1–33. 10.1075/lab.1.1.01sor 33486653

[B58] Sorace A. Filiaci F. (2006). Anaphora resolution in near-native speakers of Italian. *Second Lang. Res.* 22 339–368. 10.1191/0267658306sr271oa

[B59] Stevenson R. J. Crawley R. A. Kleinman D. (1994). Thematic roles, focus and the representation of events. *Lang. Cogn. Process.* 9 519–548. 10.1080/01690969408402130

[B60] van Dijk T. A. Kintsch W. (1983). *Strategies of discourse comprehension.* New York, NY: Academic Press.

[B61] Yang C. L. Gordon P. C. Hendrick R. Wu J. T. (1999). Comprehension of referring expressions in Chinese. *Lang. Cogn. Process.* 14 715–743. 10.1080/016909699386248

[B62] Yang C. L. Gordon P. C. Hendrick R. Wu J. T. Chou T. L. (2001). The processing of coreference for reduced expressions in discourse integration. *J. Psycholinguist. Res.* 30 21–35. 11291181 10.1023/a:1005252123299

[B63] Zhang A. Kwon N. (2022). The interpretational preferences of null and overt pronouns in Chinese. *J. Linguist.* 58 649–676. 10.1017/S0022226721000402

